# Effects of TPA023B, an α2/3/5GABA_A_ subtype-selective partial allosteric modulator, on fentanyl-induced respiratory depression in male rats: comparison with the non-selective benzodiazepine midazolam

**DOI:** 10.1007/s00213-025-06945-1

**Published:** 2025-11-01

**Authors:** Loc M. Pham, Andria B. Nanney, Jordan Hasting, Daniela Rüedi-Bettschen, Kevin B. Freeman, James K. Rowlett

**Affiliations:** Department of Psychiatry and Human Behavior, Center for Innovation and Discovery in Addictions, University of Mississippi Medical Center, Jackson, MS USA

**Keywords:** Respiratory depression, TPA023B, Midazolam, Fentanyl, Whole-body plethysmography

## Abstract

**Rationale:**

Opioid-related overdose deaths involving benzodiazepines have increased in recent years, and prior studies have reported that clinically used benzodiazepines can enhance the respiratory-depressant effects of mu opioid receptor (MOR) agonists. TPA023B is an α1-sparing GABA_A_ positive allosteric modulator developed as a potential anxiolytic with fewer benzodiazepine-typical side effects. However, it is unknown if and to what degree TPA023B can affect MOR-agonist induced respiratory depression.

**Objectives:**

The current study compared the effects of either TPA023B or midazolam, alone and combined with fentanyl, on respiratory depression in rats, using whole-body plethysmography.

**Methods:**

Male Sprague-Dawley rats were implanted with chronic indwelling intravenous (i.v.) catheters for drug infusions. Respiration (frequency, tidal volume, and minute volume) was measured using whole-body plethysmography. The tests consisted of a pretreatment (midazolam, 30 mg/kg; i.v.; TPA023B, 1.0 mg/kg; i.v; or vehicle) followed by a fentanyl injection (0.01, 0.03, 0.1 mg/kg, i.v., or vehicle) and 60 min of respiration assessment.

**Results:**

Overall, fentanyl alone, but not TPA023B or midazolam alone, produced dose-dependent reductions in tidal volume and minute volume. When administered as pre-treatments, neither midazolam nor TPA023B increased the magnitude of fentanyl-induced respiratory depression, instead producing prolonged reductions in tidal volume and minute volume. Midazolam produced a more sustained reduction in these parameters than TPA023B.

**Conclusions:**

Benzodiazepines prolong fentanyl-induced respiratory depression, but this effect may be reduced by eliminating activity at α1GABA_A_ receptor subtypes.

## Introduction

Opioids and benzodiazepines are often taken together for both therapeutic and non-therapeutic (i.e., recreational) purposes. Regarding the former, combinations of midazolam and fentanyl are used concurrently for the induction of anesthesia prior to surgical procedures. In recreational users, opioid-related overdose deaths involving fentanyl have increased in recent years, with the co-use of fentanyl and benzodiazepines rising five-fold since 2015 (National Institute on Drug Abuse 2023). Both clinical and preclinical reports indicate that conventional benzodiazepines can enhance opioid-induced respiratory depression, the most likely cause of death following these combinations (e.g., Nilsson et al. 1986; Bailey et al. 1990; Xu et al. 2020; Gershuny et al. 2025).

Consistent with this overall observation, a study in human patients reported that apnea occurred in a greater number of subjects receiving co-administration of fentanyl and midazolam than those administered with fentanyl alone (Bailey et al. 1990). However, preclinical data with respiratory effects of fentanyl combined with benzodiazepines are somewhat mixed in terms of the nature and magnitude of the combined effects. In this regard, a study in rhesus monkeys testing combinations of opioids and benzodiazepines (including fentanyl and midazolam) reported modest effects, described as “less than additive” (Gerak et al. 1998). In contrast, Xu et al. (2021) demonstrated effects on respiration measures that were additive or synergistic between diazepam and oxycodone in rats, whereas combination of midazolam with heroin potentiated heroin-induced reductions in brain oxygen use and induced hypoxia in rats (Afzal and Kiyatkin 2019). These disparate results may represent differences in species, dose ranges, and methodologies.

Little is known about the mechanisms through which MOR agonists and benzodiazepines produce enhanced respiratory depression. The pontine Kӧlliker-Fuse/parabrachial nuclei and the medulla’s preBӧtzinger-complex are the key brain regions in modulating opioid-induced respiratory depression (Del Negro et al. 2018; Ramirez et al. 2021). Research suggests that opioids and benzodiazepines bind to their receptors located in these regions, leading to the inhibition of respiratory circuits and compounding respiratory depression (Webster and Karan 2020; Dahan et al. 2022). From a pharmacological mechanism perspective, GABA_A_ receptor subtypes containing α1-subunits (α1GABA_A_ receptors) have been implicated in respiratory depression in mice (Masneuf et al. 2012). Interestingly, a study in rats that compared the respiratory effects of alprazolam to KRM-II-81, a novel compound with preferential efficacy at α2/α3-subunit-containing receptors (α2/α3GABA_A_ receptors), reported that both reduced respiration frequency (Witkin et al. 2019). However, KRM-II-81 did not significantly decrease tidal or minute volume, in contrast to alprazolam. These results raise the possibility that compounds with reduced modulation of α1GABA_A_ receptors (i.e., α1-sparing compounds) may be safer than conventional benzodiazepines and also may be predicted to engender less respiratory depression when combined with MOR agonists like fentanyl.

The present study sought to determine the potential respiratory depressant effects of midazolam, alone and combined with fentanyl in rats following i.v. administration, in order to provide additional data on the potential adverse effects of these combinations. In addition, these studies provided a comparator for respiratory experiments with TPA023B, an imidazotriazine with selective efficacy at α2GABA_A_, α3GABA_A_, and α5GABA_A_ receptor subtypes (Atack et al. 2010). Specifically, TPA023B has zero efficacy (i.e., is an antagonist) at α1GABA_A_ subtypes, and has partial PAM effects at the other three subtypes (referred to as “α1-sparing”). We hypothesized that TPA023B would have no respiratory depressant effects on its own due to it lacking activity at α1GABA_A_ subtypes, and we further predicted that TPA023B would have few, if any, effects when combined with fentanyl. All studies used whole-body plethysmography (WBP) techniques in adult, male rats. Doses of the GABA_A_ PAMs were chosen to reflect higher levels associated with recreational use. In this regard, an intravenous (i.v.) dose of 1.0 mg/kg of TPA023B was chosen as likely to have ~ 100% receptor occupancy, since this dose via the oral route occupied an estimated 87% of rat brain benzodiazepine binding sites (Atack et al. 2010). We do not have access to receptor occupancy data for midazolam, but an earlier study demonstrated LD_50_ median dose of 357 mg/kg, i.v., with a dose of 130 mg/kg, i.v., inducing severe respiratory depression without lethality (Gueye et al. 2002). We used an approximately 3- to 10-fold lower dose of midazolam, in order to avoid toxicity of this drug alone but to remain in the higher range, presumably reflecting full receptor occupancy.

## Method

### Subjects and surgical procedure

The subjects were 8 male Sprague-Dawley rats weighing between 285 and 300 g at the beginning of the study. All subjects were purchased commercially (Envigo Laboratories; New Jersey, USA), pair-housed, received *ad libitum* access to water and food (Envigo), and maintained on a dark-light cycle, with light on at 1900 and off at 0700. Testing was conducted at approximately 1200 during the dark cycle. All subjects were implanted with chronic indwelling intravenous catheters and vascular access ports as described previously (Rüedi-Bettschen and Platt 2017). All procedures followed the National Research Council’s Guide for Care and Use of Laboratory Animals (2011), and all procedures were approved by the University of Mississippi Medical Center’s Institutional Animal Care and Use Committee.

## General procedure

Respiratory measures were obtained using a Buxco whole-body plethysmography (WBP) system for rats (DSI; Minnesota, USA). Each chamber of the WBP system was configured with a pump system for automated drug delivery (Med Associates; Vermont, USA). Recorded data were processed and exported using FinePoint software (DSI). Because respiration metrics can vary as a function of body weight, the FinePoint software normalizes volumetric data based on current weight. This is a key feature of the analysis, given our rats gained weight over the course of the study at the expected rate for adult male Sprague-Dawley rats.

All tests were conducted at least 48 h apart in order to account for the relatively long elimination half-life of TPA023B (Atack et al. 2010). For each test, the subjects received an experimenter-administered pretreatment (i.v.) of either vehicle (1.0 ml/kg), TPA023B (1.0 mg/kg) or midazolam (10 or 30 mg/kg), followed by a 0.1 ml infusion of vehicle to clear the catheter of the pretreatment drug. Immediately after, subjects were placed into the chamber for a period of 90 min. Plethysmography recording began at minute 0 and lasted for the full 90 min, and there were no experimenters in the room during this period to optimize stable respiratory performance. Following a 30-min habituation period, a programmed infusion of fentanyl (0.01, 0.03, or 0.1 mg/kg; i.v.) or saline was administered. For all infusions, the pumps were programmed for approximately 20 s duration, rendering a volume of approximately 0.4 ml, depending on the weight of each subject.

With regards to an experimental timeline, all rats first were evaluated with fentanyl, midazolam, and TPA023B alone. Doses for the combination studies were chosen based on the results of the tests of drugs/compound alone. The general design of the experiment was to assess a dose-response function of fentanyl on respiratory measures, without and with one or two doses of TPA023B or midazolam, respectively. Therefore, once doses were chosen for the second phase of the study (combinations), the rats were tested with fentanyl in combination with TPA023B and midazolam, in that order. The dose-response function for fentanyl obtained in the first phase of the study was used for the analyses and was not repeated. Doses were counterbalanced across animals within each drug or drug combination condition.

## Drugs

Fentanyl hydrochloride was generously provided by the National Institute on Drug Abuse (NIDA) Drug Supply Program (Maryland, USA). Midazolam was purchased from Sigma-Alldrich (St. Louis, MO) and TPA023B was a generous gift of Merck, Sharp, and Dohme, Ltd (Harlow, Essex, UK). Fentanyl hydrochloride was dissolved in 0.9% saline. TPA023B and midazolam were dissolved in ethanol 95%: Tween 80: 0.9% saline at 1:1:8 ratio. Doses were expressed as the salt form for fentanyl but base forms for midazolam and TPA023B.

### Data analysis

The dependent measurements were frequency (breaths/min), tidal volume (ml), and minute volume (ml/min), and only data from minute 30 to 60 were taken for statistical analysis. Recorded data were exported as a chronological series of 1-min averages into GraphPad Prism 10 (Dotmatics; Massachusetts, USA). These data were analyzed in 5 separate experiments: Effects of fentanyl alone, TPA023B alone, midazolam alone, combinations of fentanyl and TPA023B, combinations of fentanyl and midazolam. These data were analyzed by separate (for each respiration parameter) one-way or two-way repeated measures ANOVA with treatment and time post-injection as factors. In addition, a priori tests were included that compared the effects of vehicle with drug treatment on breathing parameters using the Bonferroni t-statistic. We also included a measure of maximum respiratory depression (i.e., the lowest respiratory measurement irrespective of dose and time; referred to as “RD_max_”). A paired t-test (TPA023B alone) or one-way repeated-measures ANOVA and Bonferroni t-tests comparing all groups to vehicle + vehicle or vehicle + fentanyl (midazolam alone, all combination tests) was used to analyze these transformed data (note that the RD_max_ transformation was applied *a posteriori*, using the *N* = 4 rats that completed all conditions). For ANOVAs in which subject attrition occurred, we used mixed effects model (MEM) analyses. Note that MEM analysis in GraphPad Prism uses the Restricted Maximum Likelihood (REML) analysis and approximations of degrees of freedom. All statistical analyses were constrained at *p* ≤ 0.05.

## Results

### TPA023B and midazolam alone

Neither the α1-sparing GABA_A_ PAM, TPA023B, nor the non-selective PAM, midazolam altered any respiration parameter when tested alone and compared with vehicle conditions over the 30 min sampling time (see Table 1). Moreover, the RD_max_ measure, which was designed to identify decreases in respiration parameters irrespective of time and dose, thereby reducing the influence of individual variability in terms of onset time, also did not reveal any effects of TPA023B or midazolam (Table 2). Paired t-tests demonstrated no significant effect of TPA023B vs. vehicle on frequency [t(3) = 2.701, *p* = 0.074], tidal volume [t(3) = 1.262, *p* = 0.269] or minute volume [t(3) = 1.197, *p* = 0.317]. One-way repeated measures ANOVA demonstrated no significant effects of midazolam at either dose vs. vehicle, for frequency [F(2,6) = 3.248, *p* = 0.155], tidal volume [F(2,6) = 0.807, *p* = 0.451], or minute volume [F(2,6) = 0.028, *p* = 0.930].

**Table 1:** Midazolam and TPA023B do not alter respiratory parameters in adult male rats (*N* = 8 total)

Treatment	Parameter	Dose	F(dfn, dfd)^a^	*p*	Diff from veh?^b^
Midazolam	Frequency	10 mg/kg, i.v.	F(60,420) = 0.820	0.538	No
		30 mg/kg, i.v.			No
	Tidal Volume	10 mg/kg, i.v.	F(60,420) = 1.072	0.393	No
		30 mg/kg, i.v.			No
	Minute Volume	10 mg/kg, i.v.	F(60,420) = 0.787	0.555	No
		30 mg/kg, i.v.			No
TPA023B	Frequency	1.0 mg/kg, i.v.	F(30,210) = 1.021	0.443	No
	Tidal Volume	1.0 mg/kg, i.v.	F(30,210) = 1.261	0.176	No
	Minute Volume	1.0 mg/kg, i.v.	F(30,210) = 0.902	0.617	No
a. F values for the interaction of treatment x time, 2-way repeated measures ANOVAs. Numbers in parenthesis are degrees of freedom for the numerator (dfn) and the denominator (dfd) for the F ratio. b. Results from individual Bonferroni t-tests for each dose vs. vehicle for every time point post injection.					

**Table 2:** Effects of the non-selective benzodiazepine, midazolam, and the 2/3/5GABAA subtype-specific compound, TPA023B, on maximal respiratory depression in male rats (*N* = 4)

α					
	Treatment				
	Vehicle	Midazolam 10 mg/kg, i.v.	Midazolam 30 mg/kg, i.v.		TPA023B 1.0 mg/kg, i.v.
Parameter	RD_max_* (mean, SEM)				
Frequency (breaths/min)	69.4	77.9	80.1		77.9
*SEM*	*3.3*	*4.8*	*5.1*		*4.9*
Tidal Volume (ml)	1.3	1.3	1.5		1.4
*SEM*	*0.06*	*0.10*	*0.13*		*0.09*
Minute Volume (mls/min)	154.6	160.1	158.6		165.3
*SEM*	*11.0*	*16.7*	*31.0*		*8.8*
*RD_max_ is the lowest respiration parameter for individual rats, irrespective of time point. No parameter values were statistically significant from the vehicle condition.					

## Fentanyl alone

**Fig. 1:**
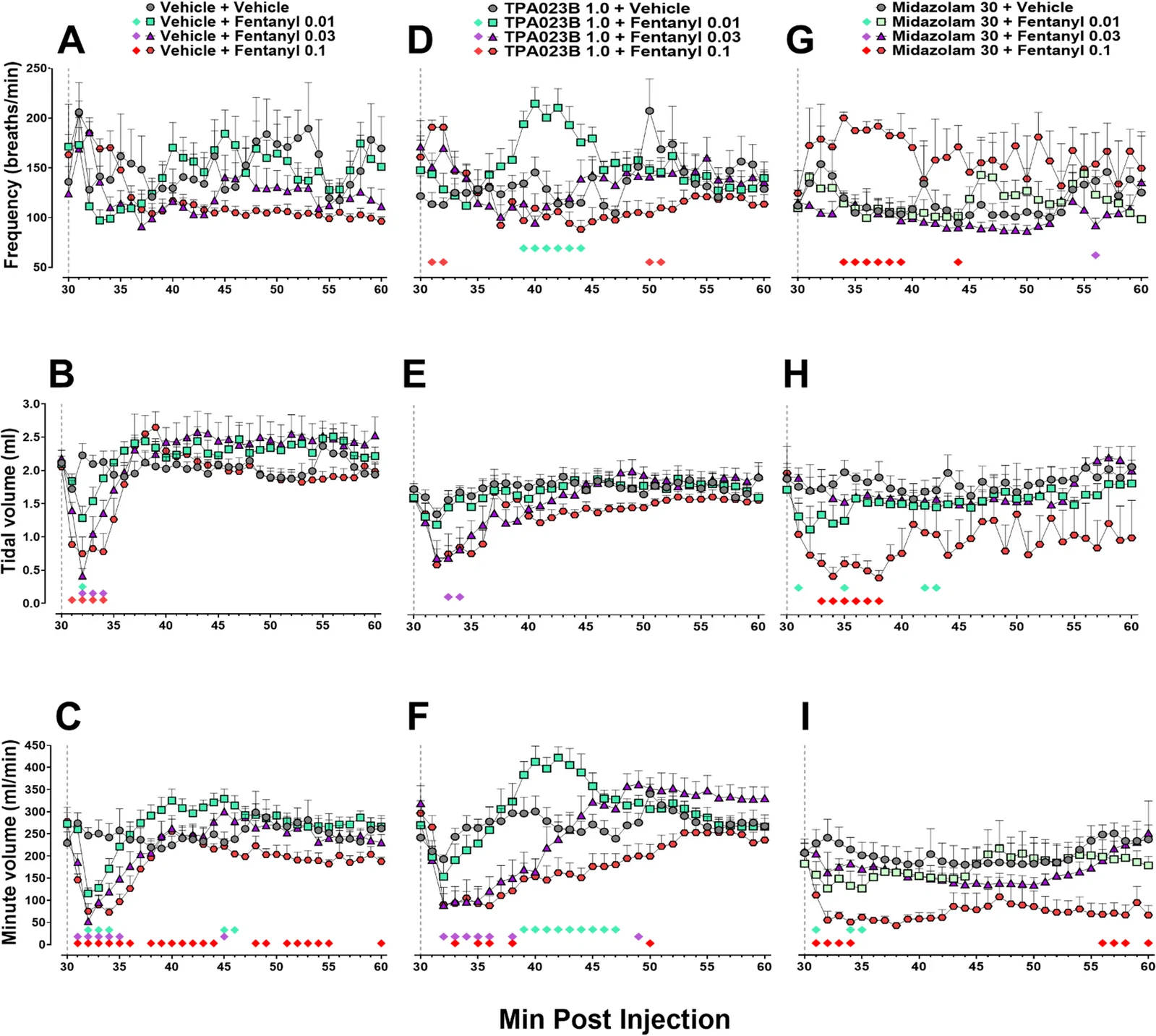
Effects of different doses of fentanyl, alone and combined with either the α1-sparing GABA_A_ PAM, TPA023B, or the non-selective GABA_A_ PAM, midazolam, in male rats. Sample sizes were *N* = 8 for vehicle + fentanyl, *N* = 7 for TPA023B + fentanyl, and *N* = 4 for midazolam + fentanyl (only 4 animals were evaluated at the highest midazolam + fentanyl combination; the analysis is restricted to these 4 rats only). Respiratory parameters (frequency, breath/min; tidal volume, mls; and minute volume, mls/min) were obtained using whole body plethysmography, in 30-min sessions (data are means ± SEM for each min during the session). The left columns show vehicle + fentanyl (i.e., fentanyl alone) for (**A**) frequency; (**B**) tidal volume; and (**C**) minute volume. The center column presents TPA023B (1.0 mg/kg, i.v.) pretreatment followed by one of three doses of fentanyl on (**D**) frequency; (**E**) tidal volume; and (**F**) minute volume. The right column shows midazolam (30 mg/kg, i.v.) pretreatment followed by one of three doses of fentanyl on (**G**) frequency; (**H**) tidal volume; and (**I**) minute volume. Vertical dashed line represents the time of i.v. injection. The colored diamonds correspond to dose of fentanyl that is significantly different from vehicle + vehicle (left columns), TPA023B + vehicle (center columns) or midazolam + vehicle (right columns), Bonferroni t-tests, p’s < 0.05

Figure 1 shows the effects of fentanyl doses alone (with TPA023B vehicle co-administration) on respiratory frequency (Fig. 1A), tidal volume (Fig. 1B), and minute volume (Fig. 1C). For frequency, only a significant main effect of drug treatment was obtained [F(3,21) = 3.920, *p* = 0.031]. More systematic effects of fentanyl were observed with tidal volume (Fig. 1B), with a significant treatment x time interaction [F(90,630) = 3.168, *p* = 0.012] and Bonferroni t-tests revealing a largely dose-dependent decrease vs. vehicle over the first approximately 5 min following i.v. infusion (p’s < 0.05). A more profound decrease was evident for minute volume [treatment x time interaction, F(90,630) = 2.544, *p* < 0.0001], with dose and time dependent decreases vs. vehicle throughout the session, especially at the highest dose tests (p’s < 0.05). Of note, significant increases from vehicle administration were observed for the two lower doses at 45 and 46 min (p’s < 0.05).

## Combinations of TPA023B with fentanyl

Panels D, E, and F of Fig. 1 show the effects of combinations of TPA023B (1.0 mg/kg, i.v. >90% occupancy dose) with the three doses of fentanyl. Figure 1D shows the complex effects of TPA023B and fentanyl combinations on frequency, in which a significant treatment x time effect was observed [MEM, F(5.090, 30.54) = 2.959, *p* = 0.0265; note that one animal lost a catheter, reducing the *N* = 7 for some but not all conditions]. Bonferroni t-tests showed that the 0.1 mg/kg dose of fentanyl early in the session, and the lowest fentanyl dose (0.01 mg/kg, i.v.) from 39 to 44 min post-infusion showed significant increases above TPA023B alone (p’s < 0.05). By 50 min post-infusion, significant decreases from TPA023B in frequency were observed with the highest dose of fentanyl (p’s < 0.05). For tidal volume (Fig. 1E), only a main effect of time was observed [MEM, F(2.869,20.09) = 10.60, *p* = 0.0002] with Bonferroni t-tests showing significant decreases from TPA023B alone at the intermediate dose (0.03 mg/kg fentanyl, i.v.) at minutes 33 and 34 post-infusion. However, considerably more complex interactions were observed with minute volume in Fig. 1F. The treatment x time interaction was significant for minute volume [MEM, F(3.882, 21.83) = 5.447, *p* = 0.0036] with Bonferroni t-tests showing suppression of minute volume by 0.03 and 0.1 mg/kg fentanyl compared to TPA023B alone early during the session, which extended to at least 50 min after the infusion. Notably, starting at around 39 min post-infusion, a robust increase in minute volume occurred between minute 38 to minute 47 engendered by the lowest dose of TPA023B tested (Fig. 1F). All effects on minute volume in combination with fentanyl appeared to have dissipated by minute 55. In summary, TPA023B, while having no respiratory depressive effects on its own, resulted in a combination of enhanced frequency and minute volume at the lowest fentanyl dose, whereas combining TPA023B with the highest dose of fentanyl engendered a prolongation of fentanyl-induced decreases in minute volume.

### Combinations of midazolam with fentanyl

Four rats were evaluated initially with this combination (we have four WBP chambers), and by the end of the session were immobile and showing tachypnea (as evidenced by low minute volume but elevated, albeit variable, breathing frequency). Only one of the four rats was successfully revived with naltrexone injections; the other rats died and testing was halted. Although other rats were tested at other midazolam + fentanyl combinations, only the four rats evaluated at the highest dose combination were used for the complete the analysis for this combination.

Panels G, H, and I of Fig. 1 show the effects of combinations of midazolam (30 mg/kg, i.v. ~ ½ log_10_ unit below respiratory depressant dose; Gueye et al. 2002) with the three doses of fentanyl. Figure 1G shows that with midazolam, the highest dose of fentanyl (0.1 mg/kg, i.v.) increased respiratory frequency above midazolam alone levels (pre-planned Bonferroni t-tests, note that the omnibus ANOVA was not significant). The Bonferroni t-tests showed that from 34 min to 44 min, the majority of mean frequencies for the highest fentanyl dose were significantly higher than those for midazolam alone (p’s < 0.05). For tidal volume (Fig. 1H), midazolam combined with the lowest and highest doses of fentanyl decreased this measure, compared with midazolam alone, at the beginning of the session (pre-planned Bonferroni t-tests, note that the omnibus ANOVA was not significant). Interestingly, the decreases in tidal volume by 0.1 mg/kg fentanyl occurred over 33 to 38 min post-infusion, approximately the same time as the increases in frequency induced by this higher dose of fentanyl.

For minute volume (Fig. 1I), there was a main effect of treatment only [F(3,270) = 6.502, *p* = 0.048]; however, the pre-planned Bonferroni t-tests showed suppression by 0.01 and 0.1 mg/kg fentanyl compared to midazolam alone early during the session, and with the 0.1 mg/kg dose at the end of the session. In summary, midazolam, while having no respiratory depressive effects on its own, resulted in a combination of enhanced frequency and reduced minute volume at the highest fentanyl dose, with the minute volume suppression lasting the entire session. The combination of 30 mg/kg, i.v., of midazolam plus 0.1 mg/kg, i.v., fentanyl proved fatal in ¾ of the rats tested, even with attempted rescue with an opioid antagonist.

### RD_max_ analysis

**Fig. 2:**
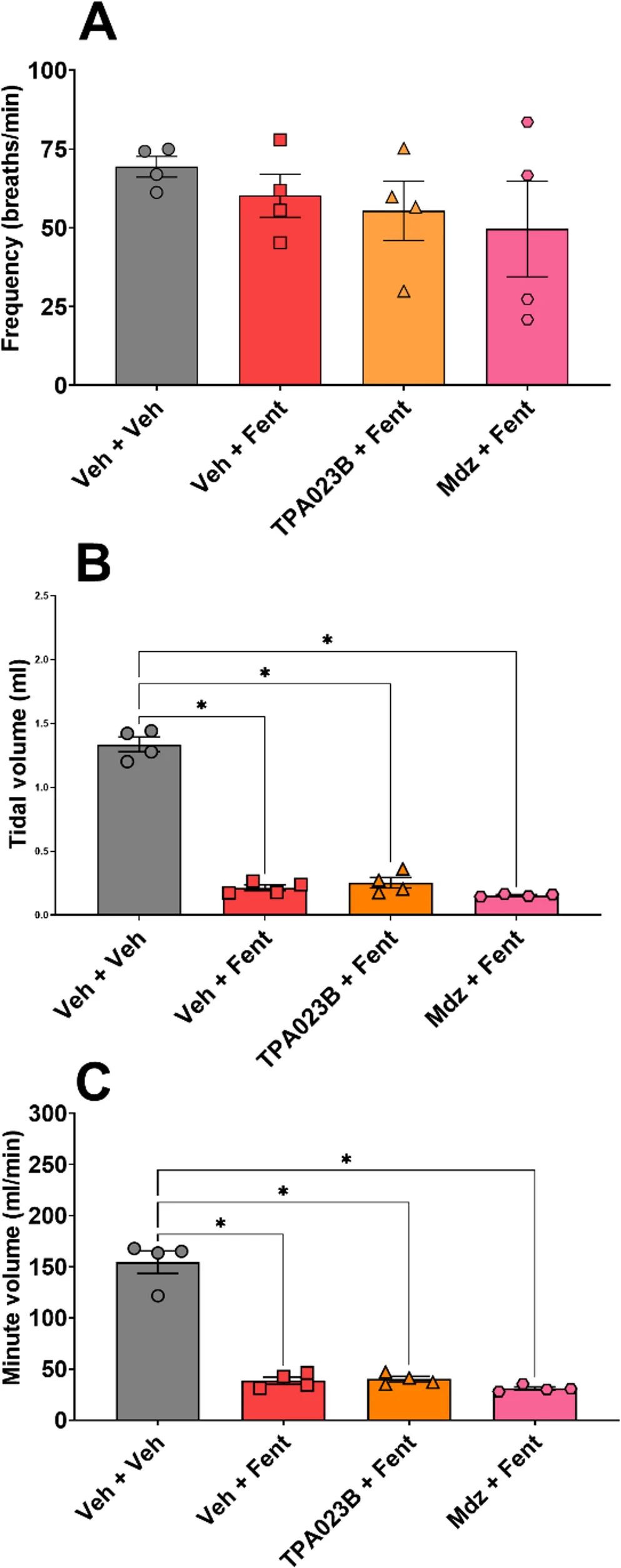
Summary measure showing maximum respiratory depression (RD_max_) for each respiratory parameter, irrespective of dose or time after injection for each rat (*N* = 4). The lines with asterisks represent significant planned comparisons using Bonferroni t-tests (p’s < 0.05). Abbreviations: Veh, vehicle; Fent, fentanyl; Mdz, midazolam. Panel A is frequency (breaths/min), panel B is tidal volume (mls) and panel C is (mls/min). All data are mean ± SEM, plus individual data points for each rat

In order to compare the magnitude of respiratory depression and to consider individual differences in each of the three respiratory parameters, we computed RD_max_, which is the lowest value of a parameter for each subject, irrespective of dose or time after infusion. For frequency (Fig. 2A), there were no effects of a one-way repeated measures ANOVA or Bonferroni t-tests, which likely reflects the fact that most significant effects on frequency were increases, rather than decreases in rate (analysis of maximum increases in frequency irrespective of dose or time revealed no significant effects, data not shown). For tidal volume (Fig. 2B), the one-way repeated measures ANOVA was significant [F(3,9) = 209.4, *p* = 0.00002], and of the pre-planned tests, vehicle + vehicle was significantly different from vehicle + fentanyl, TPA023B + fentanyl, and midazolam + fentanyl. The other comparisons were not significant (vehicle + fentanyl vs. TPA023B + fentanyl vs. midazolam + fentanyl), indicating that the magnitude of tidal volume decrease by fentanyl was neither enhanced nor reduced by combination with the GABA_A_ PAMs. Similarly, for minute volume (Fig. 2C), the one-way repeated measures ANOVA was significant [F(3,9) = 109.3, *p* = 0.0006], with pre-planned comparisons showing that vehicle + vehicle was significantly different from vehicle + fentanyl, TPA023B + fentanyl, and midazolam + fentanyl. Also, as with tidal volume, the other comparisons were not significant (vehicle + fentanyl vs. TPA023B + fentanyl vs. midazolam + fentanyl), indicating that the magnitude of minute volume decrease by fentanyl was neither enhanced nor reduced by combination with the GABA_A_ PAMs.

## Discussion

The present study evaluated the respiratory depressant effects of TPA023B, an α1-sparing GABA_A_ PAM, and the non-selective benzodiazepine midazolam, alone and in combination with the MOR agonist, fentanyl. TPA023B is representative of a novel pharmacological class of compounds currently being evaluated in clinical trials as anxiolytics and epilepsy treatments (e.g., Cerevel Therapeutic’s darigabat; Moraru et al. 2024). Benzodiazepine-type drugs often are used in combination with opioid drugs, both recreationally and in the context of surgical anesthesia. Despite these common use patterns, little is known about the pharmacological effects of benzodiazepines and opioids in combination, particularly with the newer α1-sparing compounds. In this study, we found that TPA023B had no respiratory depressant effects at a dose likely to occupy 87% or greater of benzodiazepine binding sites. Prior in vivo binding studies in male Sprague-Dawley rats showed that oral doses of 0.3, 1.0 and 3.0 mg/kg displaced 61%, 87%, and 96% of [^3^H]flumazenil binding sites in whole rat brain (Atack et al. 2010). Given that an i.v. dose of TPA023B would not require absorption via the gastrointestinal system, we anticipated that 1.0 mg/kg, i.v., TPA023B would likely occupy at least 87% of binding sites, and likely at higher levels. Similar to TPA023B, we confirmed that a relatively high dose of the conventional BZ midazolam also lacked respiratory depressant effects. Both TPA023B and midazolam, however, altered the respiratory depressant effects of fentanyl, primarily by increasing breathing frequency and suppressing tidal and minute volume at later time points during the test session. In fact, the primary effect was a prolongation of the duration of action of minute volume suppression, with neither TPA023B nor midazolam enhancing the magnitude of fentanyl’s effects. The effects of TPA023B were not as robust as that of midazolam, with the latter inducing severe tachypnea that resulted in significant mortality. Moreover, TPA023B combined with the lowest dose of fentanyl tested resulted in an increase in respiration frequency and minute volume, whereas the highest dose of fentanyl tested did not suppress minute volume during the end of the session, as it did alone and when combined with midazolam.

At present, the mechanisms of action underlying the prolongation of fentanyl’s effects on minute volume by the GABA_A_ PAMs are unknown. TPA023B acts as an antagonist at α1GABA_A_ receptor subtypes and a partial PAM at α2/3/5GABA_A_ receptor subtypes (Atack et al. 2010), whereas midazolam is a non-selective, full potentiator of all GABA_A_ receptor subtypes (Kilpatrick et al. 2007). The less robust effects of TPA023B compared with midazolam may reflect either a role for α1GABA_A_ receptor subtypes in mediating fentanyl-induced decreases in minute volume or the lower efficacy of TPA023B at α2/3/5GABA_A_ receptor subtypes. Indirect support for the latter hypothesis comes from findings that the α1GABA_A_-preferring PAM, zolpidem, in contrast to the non-selective PAM diazepam, did not enhance oxycodone-induced respiratory depression in rats measured by plasma pCO_2_ (Xu et al. 2021). While these results support the idea that efficacy differences at α2/3/5GABA_A_ subtypes accounts for the differences between TPA023B and midazolam in modulating fentanyl’s effects on minute volume, further work with compounds having higher efficacy α2/3/5GABA_A_ receptors would be needed to test this possibility.

Other properties of the two drugs that may account for the differences in their effects on respiratory depression are dose and pharmacokinetic factors. Because neither TPA023B nor midazolam had effects on respiration parameters on their own, it is difficult to equate the doses used in this study functionally. The intent was to evaluate doses that obtain high levels of receptor occupancy, and although we can’t compare in vitro potencies directly, based on different studies using similar assay parameters, TPA023B is about 30- to 100-fold more potent than midazolam (cf. Atack et al. 2010; Xu et al. 2021). Because the dose of TPA023B likely occupied ~ 100% benzodiazepine binding sites (Atack et al. 2010), we assumed a similar level with midazolam, with the caveat that occupancy data for midazolam are unavailable. In terms of pharmacokinetics, although midazolam is shorter-acting than TPA023B (Atack et al. 2010), both compounds have durations of action that far exceed the session length. However, it is possible that both compounds increased the duration of fentanyl’s respiratory-depressant effects via competition with a common enzyme during metabolism. Fentanyl is primarily metabolized by CYP3A4 (Bird et al. 2023), which is also a substrate for midazolam and TPA023B metabolism (Li et al. 2021; Bird et al. 2023). Depending on the relative potencies of these ligands at CYP3A4, they could prevent metabolism of fentanyl, resulting in the prolonged effects across the session. If this is the mechanism, a critical next question is to determine the extent to which the effects of midazolam on fentanyl-induced respiratory depression generalize to other commonly-used benzodiazepines.

Also of interest from a PK perspective is the long documented multi-phasic distribution of fentanyl, postulated to be due to (a) re-distribution of fentanyl into peripheral tissue, in particular stomach tissue, in which the drug re-enters circulation via excretion into the GI system, or (b) re-distribution into circulation following activation of skeletal muscle, in which fentanyl accumulates (Bird et al. 2023). In the present study, it is notable that for minute volume, moderate effects of fentanyl alone at the highest dose were still evident at approximately 20 min post infusion (i.e., at 50 min post injection), which correlates to maximal levels of fentanyl in stomach after i.v. administration in rats (Stoeckel et al. 1979; Hug and Murphy 1981). However, after 20 min post infusion of the highest dose of fentanyl combined with midazolam, robust decreases in minute volume were evident, which likely contributed to the mortality observed after the session. The mechanism underlying this relatively delayed effect of fentanyl alone is unknown (for review, see Bird et al. 2023). However, if redistribution to the gastro-enteric system is involved, midazolam may have enhanced this delayed effect of fentanyl by slowing gastrointestinal motility, which benzodiazepines have been reported to do (Iwata et al. 1980). When considering that midazolam may also interfere with fentanyl metabolism through enzyme inhibition, the addition of a prolonged presence of fentanyl in the gastro-enteric system could significantly elevate risks of toxicological effects.

A key difference between midazolam and TPA023B was that combination with the latter compound did not result in suppressed respiration at 20 + min post infusion, and did not result in mortality. These results raise the possibility that TPA023B had some protective effect at the longer time points, although mechanism(s) of action of this effect are unclear. In this regard, there is no known mechanism by which TPA023B would alter either gastro-enteric or skeletal muscle recirculation directly. However, TPA023B has reduced sedative-motor effects compared with conventional benzodiazepines (e.g., Atack et al. 2010), which may account for the lower propensity to impair respiration, although it is important to note that the reduced sedative-motor effects are believed to be centrally mediated via this compound’s lack of efficacy at the α1GABA_A_ receptor subtype (Atack et al. 2010; Duke et al. 2018).

Although our findings raise the possibility of pharmacokinetic mechanisms underlying the respiratory effects of fentanyl + GABA_A_ PAM combinations, another study evaluating a different combination of opioid and benzodiazepine does support a pharmacodynamic (and not pharmacokinetic) explanation of the combined effects of the drugs. Vodovar et al. (2022) evaluated the partial MOR agonist, buprenorphine, combined with the non-selective benzodiazepine, diazepam, on various measures of respiratory function and brain levels of buprenorphine in rats. In general, these authors demonstrated that combining doses of buprenorphine and diazepam that were inactive when tested alone resulted in significant respiratory depression (note that buprenorphine alone, unlike fentanyl in the present study, had few effects on respiration), yet buprenorphine levels in brain were unaffected by combination with diazepam. Vodovar et al. (2022) postulated that the respiratory depression that occurred as a result of combining these two drugs was due to a complex interaction of effects on oropharyngeal, intercostal, and diaphragm muscle activity. These mechanisms certainly could play a role in fentanyl + GABA_A_ PAM interactions, with the caveat that buprenorphine is a long-acting partial MOR agonist compared with fentanyl being a short-acting full MOR agonist. Moreover, diazepam metabolism is relatively more complex than midazolam metabolism, with the former having several active metabolites (Riss et al. 2008).

Prior studies examining combined administration of fentanyl and midazolam have not reported respiratory depression as robust as that observed in the current study. Gerak et al. (1998) reported that in rhesus monkeys, midazolam produced modest leftward shifts in the fentanyl minute volume dose-response function, describing this effect as less-than additive, i.e., infra-additive. However, taking species differences into account, the dose of midazolam used in that study was considerably lower than the dose tested in the current study, although it is also noteworthy that midazolam alone induced mild respiratory depression in rhesus monkeys (Gerak et al. 1998) whereas it had no effects in the present study. While both species differences and potential methodological variances (e.g., different routes of administration, constrained vs. non-constrained subjects) likely play a significant role in the differences in results between the two studies, it also may be the case that lower doses of fentanyl have no significant effect on respiration when combined with lower doses of midazolam, consistent with clinical practice of using these drugs together. In this regard, in human subjects, a low dose of fentanyl (2.0 µg/kg, i.v.) combined with a low dose of midazolam (0.05 mg/kg, i.v.) did not result in a greater decrease in fentanyl-induced suppression of minute volume, and also did not prolong this suppression (Bailey et al. 1990). However, Bailey et al. (1990) did show that combinations of fentanyl and midazolam resulted in an increase in hypoxic events from 50% with fentanyl alone to 92% with the combination, suggesting that the interactions between fentanyl and midazolam in regulating respiration are complex and may be markedly affected by dose, methodology used, species, environmental conditions, and so on.

A caveat of this study potentially is the use of an ethanol-based vehicle for the i.v. injections of TPA023B and midazolam. This vehicle was one of the few suitable for i.v. injections of TPA023B, which is insoluble in aqueous solution. Although ethanol is a GABA_A_ PAM, the highest dose any rat received from this vehicle was 0.1 g/kg, which is well below the level of doses shown to alter opioid- or benzodiazepine-induced respiratory depression (e.g., Van Gorder et al. 1985; Kiyatkin and Choi 2024). Moreover, we did not see an effect of midazolam or TPA023B alone when using this vehicle, and the effect of fentanyl without vehicle pretreatments was no different than with vehicle pretreatments. Nevertheless, we cannot completely rule out the possibility that the relatively low dose of ethanol contributed to the effects of the combinations of midazolam or TPA023B with fentanyl, and interaction studies with ethanol are of interest, given the prevalence of ethanol use in patients misusing opioids and/or benzodiazepines.

To date, information on the effects of opioids and benzodiazepines from preclinical studies has been limited. In studies using self-administration and place preference conditioning procedures, combining benzodiazepines with opioids typically enhanced the reinforcing/rewarding effects of either drug, even if the benzodiazepine had no effect on its own (Ma et al. 2014; Weed et al. 2017; Berro et al. 2022). In contrast, Gerak et al. (1998) demonstrated no interactions between benzodiazepines and opioids in drug discrimination procedures in monkeys, suggesting no combined effects on interoceptive stimuli between the two drug classes. Nonetheless, these findings with addiction models provide some behavioral evidence consistent with an enhanced probability of the two drug classes being used together for recreational purposes. Critically, an understanding of respiratory effects of these combinations is necessary to address the enhanced toxicity associated with this complex form of polydrug addiction.

## Data Availability

All data available upon request to the corresponding author or his designees.
